# Effect of Sand Particle Size on Microstructure and Mechanical Properties of Gypsum-Cemented Similar Materials

**DOI:** 10.3390/ma13030765

**Published:** 2020-02-07

**Authors:** Weiming Guan, Qi Qi, Zhiyi Zhang, Senlin Nan

**Affiliations:** 1 College of Geology and Mines Engineering, Xinjiang University, Urumqi 830046, China; gwmxju@xju.edu.cn (W.G.); xjuzhiyi@163.com (Z.Z.); xjunansenlin@163.com (S.N.); 2 State Key Laboratory of Coal Resources and Safe Mining, School of Mines, China University of Mining and Technology, Xuzhou 221116, China; 3 Autonomous Region Experimental Teaching Demonstration Center for Geology and Mining Engineering, Xinjiang University, Urumqi 830047, China

**Keywords:** sand particle size, similar materials, gypsum cement, hydration reaction, numerical analysis, mechanical strength

## Abstract

To identify mechanism of sand particle size effect on the mechanical properties of gypsum cement, 11 grades of sand particles with a size of 0.1–3 mm were used to produce 99 specimens for uniaxial compression and permeability coefficient testing. Based on this, the distribution characteristics of internal stress and horizontal displacement are discussed using the numerical analysis. The results obtained show that the sand particle size effect on the uniaxial compressive strength of similar materials is negatively correlated within the range from −16.51% to 49.79%. SEM observations imply that, in the case of small particle sizes, gypsum crystals develop into denser needle-like structures, while for larger particle sizes, they are mostly loose lamellar structures. Permeability tests indicated that the larger the sand particle size, the greater the permeability, indicating that the internal pore connectivity is better, and the crevices are easier to penetrate when the specimen is compressed. Numerical simulations indicated that the larger the particle size, the larger the extreme deformation value of the specimen in the horizontal direction, and the more uneven the deformation distribution. In addition, specimens with larger particle sizes had a larger total area, where the tensile stress exceeded the ultimate tensile strength, and were more prone to tensile failure.

## 1. Introduction

**Figure 1:**
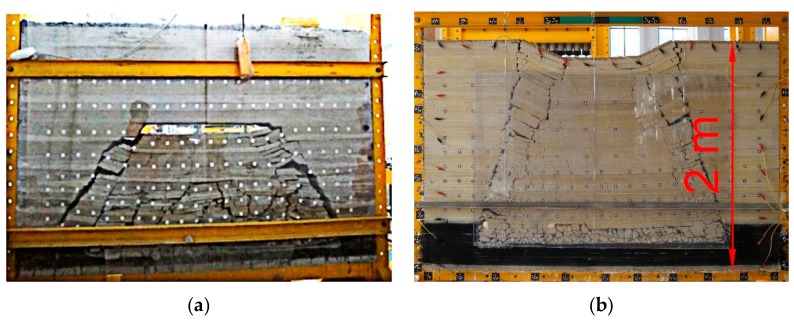
Results of similar experiments performed on desert aeolian sand in Urumqi, Xinjiang (**a**) and river sand in Xuzhou, Jiangsu (**b**).

Similar simulation experiments are important research method commonly used in geotechnical engineering, in particular, in coal mining. Thus, they have been used to study the influence of underground mining on slope movement [1], coal mining subsidence in mining area after coal mining [2,3], development pattern of mining fissures [4,5], overburden movement pattern of coal seams [6,7,8], coal and gas outburst [9,10], and so on. However, many influencing factors affect mechanical test results, which implies instability of mechanical properties of rock-soil similar materials and low test repeatability. Figure 1a,b depicts similar simulation experiments, which were conducted using the same equipment and scheme, but different types of sand, respectively: (1) Desert aeolian sand, in Urumqi, Xinjiang University of China), and (2) river sand in Xuzhou, China University of Mining and Technology. In the experiment in Xuzhou, the fissures penetrated the ground, and the main critical layer was broken and fractured. However, in the experiments in Urumqi, the main critical layer was not broken. These differences can be attributed to several influencing factors, including sand particle size, compaction degree, curing temperature, curing humidity, etc. In order to improve the accuracy and repeatability of similar experiments, it is necessary to study these factors, explore the mechanism of their effect on mechanical properties, and elaborate a more standardized experimental method.

At present, there are many achievements about similar materials of rock-soil. Most research directions are in material ratio and process error analysis. According to the different cementing composition of similar materials, they can be divided into cement/cement [11], gypsum cement [12], iron crystal sand cement [13], and other forms. Some scholars have also developed new geotechnical similar materials, according to the special needs of rockburst [14] and coalbed methane [15]. Different material ratios have a significant effect on the strength of similar materials [16]. In terms of the experimental error analysis, Luo [17] analyzed the source of strength deviations of similar materials under uniaxial compression.

Related studies have found that the particle size of sand has a great effect on the strength of cemented similar materials [18,19]. Some studies consider that the increase of small sand particles can improve the mechanical properties of cementitious materials to a certain extent [20,21]. Jinxiao Jia believed that the smaller the particle size is, the fewer defects on the surface, so the mechanical strength increases with decreasing particle size [22]; On the contrary, Haifei Lin found that the larger the sand size is, the larger the contact area between the sand particles is. The friction increases with the increase of the contact area. Therefore, the material strength can be enhanced [23]. However, the research of Jiang yu Wu has mentioned that the increase of small sand particles will lead to the deterioration of microstructure, while the pores between the larger sand particles will not be fully filled by hydrate; when the gradation is appropriate, the cemented materials has relatively dense microstructure and superior structural performance [24,25]. In summary, the influence mechanism of aggregate size on the strength of cemented materials is still very vague [26].

Consequently, this paper studies the variation of axial compressive strength of gypsum-cemented similar materials under 11 different particle size conditions. Furthermore, SEM observations and permeability experiments were conducted. Finally, the related numerical models were established.

The main contributions of the study are as follows: Firstly, the effect of particle size on mechanical properties of gypsum-cemented similar materials was obtained, which provided basic support for improving the experimental accuracy of similar simulation of rock-soil. Secondly, the development characteristics of gypsum crystals and the evolution of pore structure in the specimen were determined, which revealed the variation mechanism of the specimen mechanical strength. Finally, the internal deformation characteristic and stress distribution of the specimen were explained, which clarified the changing characteristics of the internal mechanical environment of the cemented material under pressure.

## 2. Experimental

### 2.1. Ratio of Tested Materials

It is common practice to scale down the mine strata to a lab level model, so the model and the prototype should keep a certain ratio relationship. According to the similarity theory, the ratio can be divided into three categories: Geometric similarity ratio, kinematical similarity ratio, and dynamic similarity ratio. Geometric similarity requires the length, width, and height to maintain a certain ratio. Kinematical similarity requires the corresponding points to be similar in both the model and prototype and the exercise time to maintain a certain ratio. Dynamic similarity requires all the forces between the model and the prototype to be similar and meet the following condition [27]:

The geometry, material composition, and load applied follow the similarity theory

α_p_ = α_r_ × α_l_ (1)

where α_p_ is mechanical properties similarity ratio, α_r_ is bulk density similarity ratio, and α_l_ is geometric similarity ratio.

The object of this simulation experiment was to acquire the movement and fracture law of 460 m thick mines strata under the influence of mining, and the height of the similar simulation test stand (steel frame as shown in Figure 1) was 2 m, so the geometric similarity ratio α_l_ = 2 m/460 m = 1/230 can be determined. Setting the bulk density ratio to α_r_ = 1/1.5 and substituting it into Equation (1) can get the mechanical properties similarity ratio α_p_ = 1/1.5 × 1/230 = 1/345. The average uniaxial compressive strength of the mudstone in the formation measured by experiments was 34.6 MPa. Therefore, it can be obtained that the uniaxial compressive strength of the similar material was 34.6 MPa/345 = 100.2 kPa.

Similar simulation experiments used fine sand, CaSO_4_·0.5H_2_O, and CaCO_3_ as test materials, which were mixed with water and stirred. Sand accounted for 70% of total mass, 21% was gypsum, and 9% was calcium carbonate. Water accounted for 1/9 of the total mass. Other specific data are given Table 1.

### 2.2. Experimental Scheme and Process

**Figure 2:**
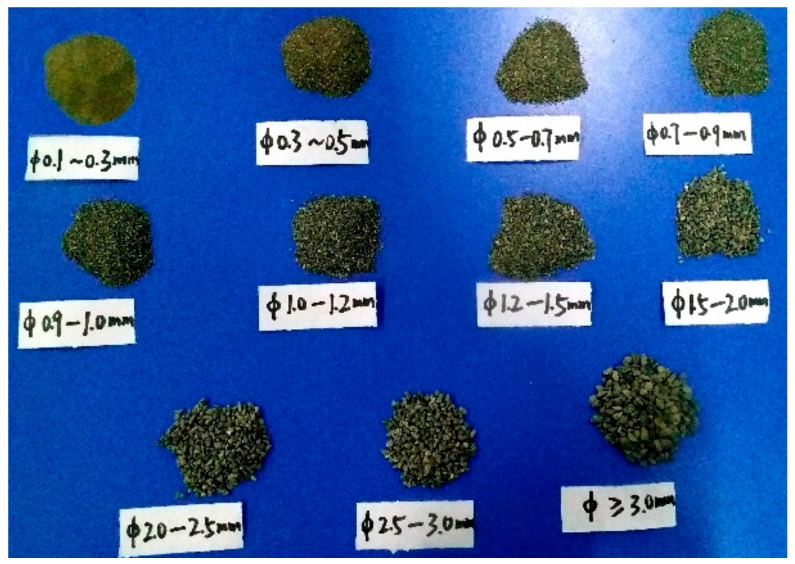
Subdivision of sand particle sizes into 11 groups.

This experiment adopts the single factor control method. In order to ensure that the sand particle size of similar simulation materials is similar to the internal structure size of the rock, excessively large sand particles were excluded from the simulation test. Meanwhile, sand particles below 4.0 mm were selected and sieved with 0.1, 0.3, 0.5, 0.7, 0.9, 1.0, 1.5, 2.0, 2.5, and 3.0 mm meshes. The respective 11 particle size ranges and groups are depicted in Figure 2.

The experimental mold was a cylinder with a size of Φ50 mm × 100 mm, which was used to produce 99 specimens (9 specimens per each group of 11). Using the electronic balance weighing, it was ensured that masses of fine sand grains, CaSO_4_·0.5H_2_O, and CaCO_3_ in each specimen were the same, which implied the identical values of their density and porosity ratio. All of the above test specimens had the same ratio, made in the same batch and on the same day, and the maintenance conditions were the same (natural indoor environment with a humidity of 30–40% and a temperature of 20–23°C), so as to reduce possible discrepancies.

## 3. Analysis of Test Results

**Figure 3:**
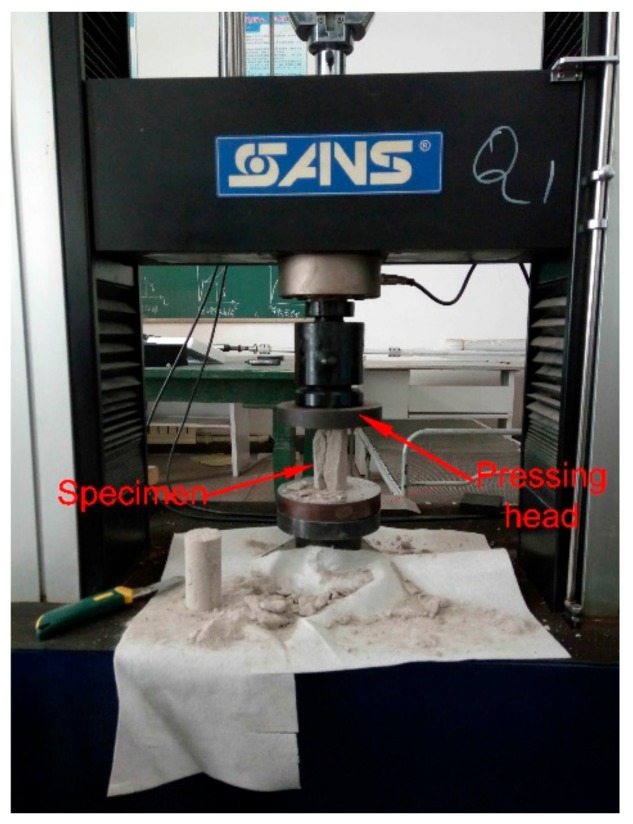
CMT5305 rock mechanics test system.

CMT5305 rock mechanics test system (MTS Industrial Systems (China) CO., LTD, Shen Zhen, China) was used for uniaxial compression test, as shown in Figure 3. And the axial load rate is 0.002 mm/s. The results of the axial compression tests are shown in Table 2. After 3, 7, and 10 days, the ultimate compressive strength of specimens was negatively correlated with the sand particle size. The average ultimate compressive strength values obtained at different curing times were 74.07, 181.13, and 228.11 N, respectively. The specimen strength stabilized after 10 days. Given that the calculated compressive strength value was 100.2 kPa, the particle size of sand strongly affected mechanical properties of similar materials. The range of compressive strength deviation from the calculated one under different particle size conditions was between −16.51% and 49.79%. When the sand particle size was between 1.5 and 2.0 mm, the experimental and calculated uniaxial compressive strength values nearly coincided.

## 4. Influence Mechanism Analysis

### 4.1. Chemical Mechanism 

#### 4.1.1. Hydration Reaction Mechanism

The hydration reaction of gypsum materials plays an important role in the mechanical properties of similar materials. The hydration reaction product can be subdivided into two parts: One is the new product produced after the reaction; the other is the residue that is not completely reacted or has not participated in the reaction. Among them, the determinant is the new product. Its shape, size, and spatial distribution determine the strength of the similar material.

The cementing material used in this experiment was CaSO_4_·0.5H_2_O, which continuously dissolved in water and underwent the hydration reaction that generates CaSO_4_·2H_2_O:CaSO4·0.5H_2_O + 1.5H_2_O→CaSO_4_·2H_2_O. Because the saturated solubility of CaSO_4_·2H_2_O (20 °C, 2.04 g/L) was much lower than that of CaSO_4_·0.5H_2_O (20 °C, 8.85 g/L), CaSO_4_·2H_2_O precipitated out of supersaturation in the solution of CaSO_4_·0.5H_2_O by uniform nucleation and growing. The equilibrium concentration of CaSO_4_·0.5H_2_O then decreased, causing CaSO_4_·0.5H_2_O to dissolve, and increased the CaSO_4_ concentration in the liquid until it reached the peak value. The CaSO_4_·2H_2_O crystal then reached the critical size, and thus, the crystallization was completed.

The crystallization of gypsum can be subdivided into two processes: Crystal nucleus production and crystal development. Different relative supersaturation is the driving force of the crystallization process, which determines the distribution density of crystal nuclei in the solution and then affects the crystal morphology. Generally speaking, the crystal forms of CaSO_4_·2H_2_O are needle-like, rod-like, plate-like, sheet-like, layered, etc. When the supersaturation is low, the distribution density of crystal nuclei is also low. The crystal grows on the nuclei, and the final single crystal has a large volume. Conversely, when the supersaturation is high, the production rate of crystal nuclei increases rapidly. The distribution density of crystal nuclei increases sharply, and the single crystals formed are smaller in volume.

#### 4.1.2. Hydration Reaction Analysis

**Figure 4:**
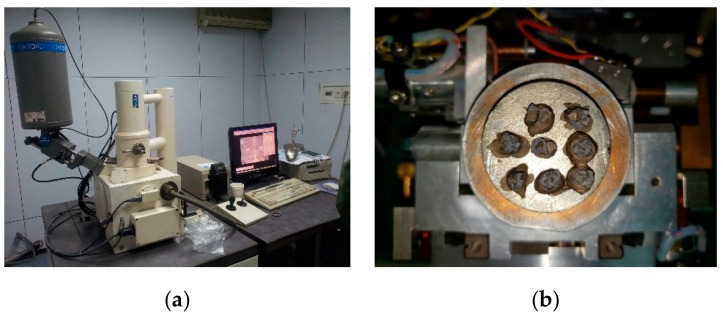
ZEISS LEO-1430 VP SEM system. (**a**) Scanning system; (**b**) Test samples coated with conductive film.

The mechanical properties of gypsum after the hydration reaction mainly depend on the developed crystal shape and the characteristics of contact points. Therefore, the ZEISS LEO-1430 VP scanning electron microscope (Carl Zeiss AG, Jena, Germany) was applied to observe microstructure and crystal shape of the gypsum-cemented similar material. However, the similar rock materials showed poor conductivity, so we needed to coat a conductive film on the surface of the material, as shown in Figure 4.

**Figure 5:**
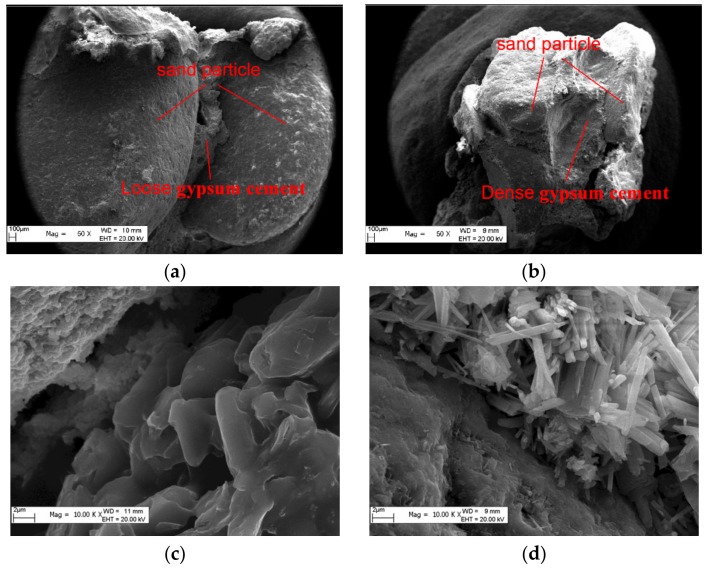
SEM crystal images of specimens with different sand particle sizes. (**a**) 60X SEM image of 2.5 mm particle; (**b**) 60X SEM image of 1.0 mm particle; (**c**) 10K X SEM image of 2.5 mm particle; (**d**) 10K X SEM image of 1.0 mm particle.

SEM observation results of a specimen with a larger sand diameter of 2.5 mm with a 60× magnification, as shown in Figure 5a, indicate that, after the hydration reaction, the sand particle structure is not dense enough, and the hydration cement product is loose, resulting in reduced specimen strength. As shown in Figure 5b, for the specimen with a smaller sand diameter of 1.0 mm, the hydration products between sand particles are denser and induce higher strength values. SEM observation results on hydration products between sand particles at the magnification of 10 K times are shown in Figure 5c: When the sand particle size was large, gypsum crystals developed into flake, layer, and plate shapes. These structures were relatively loose, which reduced the mechanical strength of the specimen. However, as shown in Figure 5d, when the sand particle size was small, needle-shaped and short, thick, rod-shaped crystals developed. The needle-shaped crystals could intertwine with each other to effectively overlap, thereby improving the shear strength of the specimen. The compressive strength of the specimen was also increased when the crystals were rod-shaped, because these were lined up orderly. The intertwinement of two crystals could produce higher overall strength.

The presence of crystal nuclei is the prerequisite for crystal growth. As it was already mentioned, all specimens in this study had the same density. When the sand particle size was small, the number of sand particles in the specimen was large, and the number of spaces formed between the sand particles was also large. The volume of a single void was small, and crystals between different pores could intertwine with each other. There were more crystal contact points per unit volume, and thus, the strength of the specimen was higher. Vice versa, when the sand particle size was large, there were fewer sand particles per unit volume, the number of pores formed between sand particles was smaller, and the volume of a single void was large. The crystals developed under the hydration reaction of pores were less prone to intertwine with each other and there were fewer crystalline contact points per unit volume, which reduced the specimen strength.

### 4.2. Physical Characteristics

#### 4.2.1. Structural Feature Analysis

**Figure 6:**
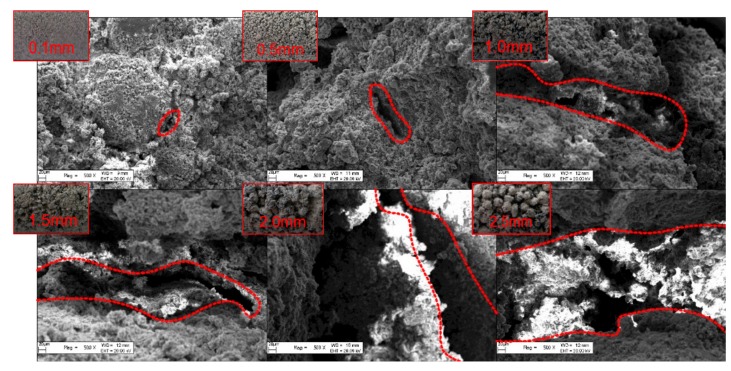
Microstructural characteristics of specimens.

As shown in the upper left corner of Figure 6, pores on the specimen surface became larger as the sand particle size increased. Interface crevices formed between different pores, and the volume of interface crevices of specimens with large pores increased accordingly. With an increase in the specimen applied load, interface crevices at the interface between the sand and cement cracked and expanded to the entire interface. When the load was further increased, the interface crevices penetrated the cement and coalesced with surrounding cracks, leading to the specimen failure. When the sand particle was large, the length and width of interface crevices in specimens were larger, as well as the probability of presence of defects in the specimen, which led to the reduction of the uniaxial compressive strength.

In order to investigate the internal microstructure of specimens, the cores of 0.1, 0.5, 1.0, 1.5, 2.0, and 2.5 mm specimens were subjected to SEM observations at magnification of 0.5 K. As shown in Figure 4, the specific surface area of sand was larger for the specimen with a smaller sand particle size. In the internal structure of specimens, the number of contact points between sand particles per unit volume increased, the contact area became larger, and the stress distribution was more uniform. The stress concentration at the interface was less probable, and thus, the specimen was less likely to be damaged. As the particle size of the sand increased, the microscopic pores formed between sand particles inside the specimen became larger. Under the same external force, the path of the force chain was singular, and it was easy to cause stress concentration that would cause damage to the specimen and reduce the material strength. 

#### 4.2.2. Pore Characteristics Analysis 

**Figure 7:**
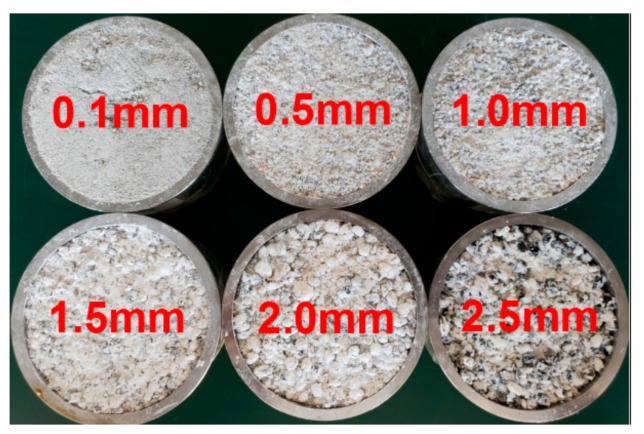
Determination of the permeability coefficient.

In order to quantitatively explore the pore characteristics of specimens, as shown in Figure 7, sand particles with sizes of 0.1, 0.5, 1.0, 1.5, 2.0, and 2.5 mm were asserted into the ring knife of the TST-55 disc permeameter (Nanjing Soil Instrument Factory Co.,Lid, Nanjing, China), using the method introduced in Section 2.1. Through the constant water pressure test, the permeability coefficient K of the sample was measured, and the pore characteristics inside the specimen were evaluated indirectly.

**Figure 8:**
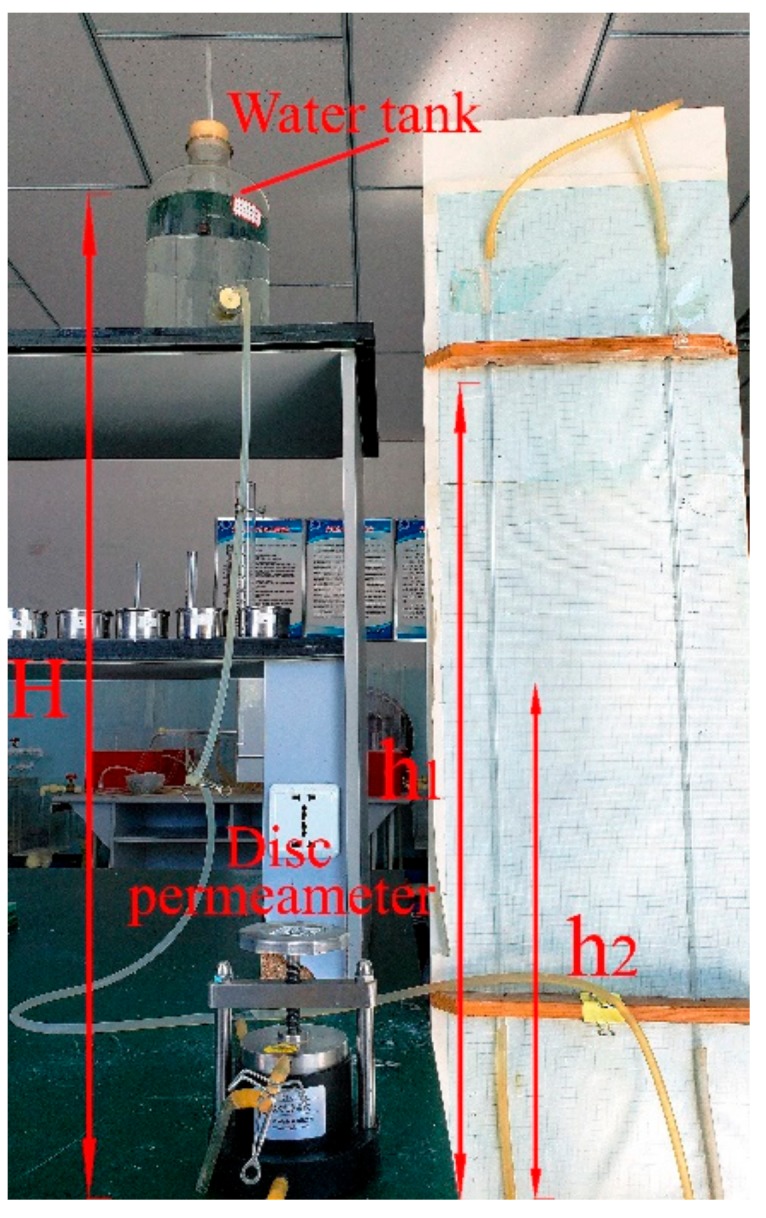
TST-55 disc permeameter.

The permeability coefficient K indicates the difficulty of the fluid passing through the pore skeleton, which equals the ratio of seepage velocity V and hydraulic gradient J. The hydraulic gradient J refers to the mechanical energy consumed by the flow through the unit length of seepage path to overcome the friction resistance; the seepage velocity V refers to the flow of the water through the unit cross-section area of the rock and soil. In the permeability test, the height of the sample is recorded as h_i_, the sectional area of the sample is recorded as s, the water level difference between the water tank and the disc permeameter is recorded as H, the liquid level is recorded as h_1_ at the beginning of the test, the liquid level reaches the height of h_2_ at the end of experiment, the time of the stopwatch is recorded as t, and the amount of overflow water is recorded as Q. The TST-55 disc permeameter is shown in Figure 8. The test results are shown in Table 3 and the permeability coefficient K was calculated by Equation (2).

K = V/J = Q × h_i_/[s × t × (h_1_/2 + h_2_/2 + H) × 10] (2)

It can be seen that the permeability coefficient of similar materials increased with the sand particle size. The magnitude of the permeability coefficient mainly depended on the size and connectivity of pores. This shows that the connectivity of pores in the specimen with a large sand particle size was strong, and the volume of a single pore was large. Due to the same porosity ratio of all specimens, the number of voids was small, and thus, stress concentration was more likely to occur when external force is applied, which could cause the specimen to fail. On the contrary, specimens with smaller sand particle sizes had smaller volumes of single pores. Under the same porosity ratio, there were more voids with worse coalescence and more uniform distribution, which impede the stress concentration and improve the specimen mechanical strength.

## 5. Numerical Analysis

### 5.1. Model Definition

It is difficult to observe the internal deformation characteristic and stress distribution of the specimen in uniaxial compression test, but that is easy to be clarified through the numerical simulation. The numerical analysis result will also be conducive to understand the inherent mechanism of the particle size effect on mechanical properties of gypsum-cemented similar materials.

The numerical models of specimens with two (large and small) particle sizes were established using the COMSOL Multiphysics software package. The rectangular geometry of the two-dimensional model was used with reference to the maximum cross section of the specimen (100 mm in height and 50 mm in width). This rectangle was filled with circles, which represented sand particles. The model of large sand particles (of 5 mm in radius) contained 50 sand particles in total, while that of small sand particles (with radius of 2.5 mm) contained 200 sand particles. The total area of the sand particles was 1250π mm^2^, which ensured that the density and porosity ratio of both specimen models were the same.

The elastic moduli of sand and cementitious material were 20 GPa and 5 GPa, and their Poisson’s ratios were 0.1 and 0.2. For axial compression, the sample was compressed by giving a prescribed displacement to the top boundary, and gradually increased the vertical displacement, by means of parametric sweep (step length was 0.002 mm). This allowed the right and left boundaries to expand freely in the radial direction and applied a fixed constraint at the bottom boundary.

In order to make the model solution converge and reduce the simulation time, a necessary simplification of the established model was performed. The assumptions of the model were as follows: (1) It was assumed that both sand particles and cementitious material were isotropic homogeneous bodies, which were uniformly distributed in the specimen. (2) Sand particles were assumed to be circular, and the diameter of each sand particle in the same specimen was the same. (3) In order to ensure a uniform loading of external loads, non-end regions were randomly selected as pores.

### 5.2. Random Distribution of Pores

**Figure 9:**
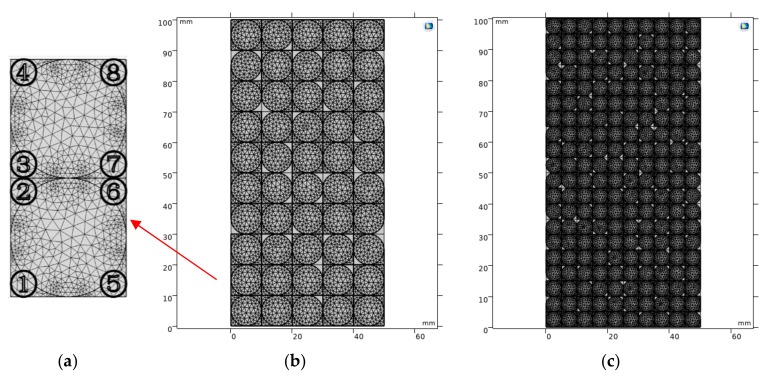
Random pore model. (**a**) Serial number of pores; (**b**) The distribution of pores in large particle model; (**c**) The distribution of pores in small particle model.

From the experimental observation of the internal and external structures of gypsum-cemented similar simulation material specimens, it is seen that the cement produced by the hydration reaction of gypsum inside the specimen did not completely fill all the pores. As shown in Figure 9a, to generate a randomly distributed pore model, the four areas around a single sand grain can be classified. Therefore, according to the geometric characteristics, it can be concluded that the surrounding areas of large and small sand particles were referred to as 1–200 and 1–800, respectively. Among them, 20% of the area was randomly selected as pores. Therefore, 40 areas with large particle sizes and 160 areas with small particle sizes were used to simulate the distribution of pores in the specimen after the hydration reaction of gypsum. Other areas are set as the properties of gypsum cement, as shown in Figure 9b,c. In order to improve the accuracy of numerical experiments, three sets of random models were set up for analysis.

### 5.3. Numerical Simulation Results and Analysis

It is difficult to obtain accurate mechanical parameters, because of the complex mechanical properties of similar rock material. Therefore, it should be noted that this numerical model can only explain the change trend, and it cannot be quantitatively compared with physical experiments.

#### 5.3.1. Analysis of the Deformation Pattern of the Specimen

**Figure 10:**
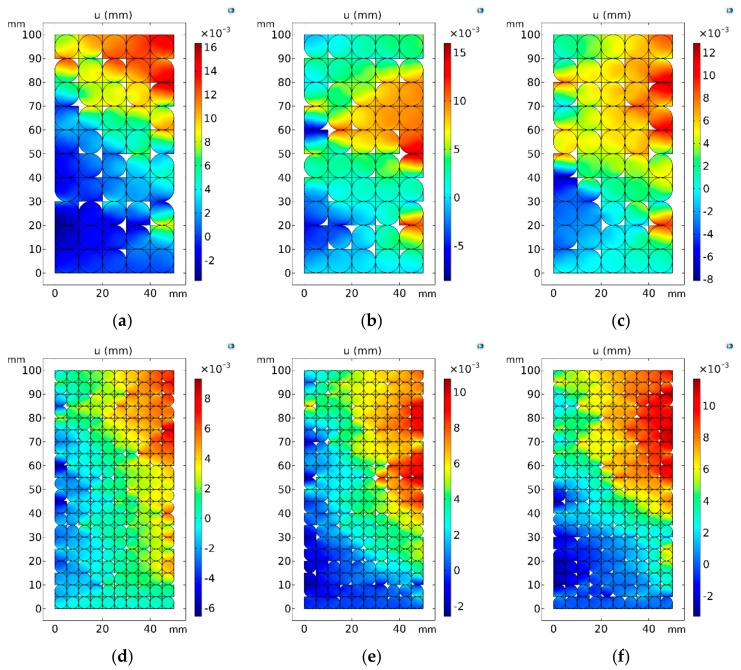
Distribution characteristics of horizontal displacement of specimens. (**a**–**c**) Distributions of horizontal displacement of large particle samples in three random simulation experiments; (**d**–**f**) Distribution of horizontal displacement of small particle samples in three random simulation experiments.

The specimen undergoes radial deformation after compression. The larger the deformation, the more likely is its failure. Therefore, the influence of the particle size on the specimen strength can be compared by analyzing the horizontal displacement. To this end, the same velocity boundary was applied at the top of the model, and the horizontal displacement of the two groups of specimens is compared at the same time. It can be seen from Figure 10 that the horizontal displacement distribution of each specimen appeared positive and negative, indicating that the specimen exhibited expansive deformation. In addition, the negative horizontal displacement was mostly distributed in the lower left part of the specimen, while the positive displacement was distributed in its upper right part. From this, it can be inferred that the form of the fracture was mostly a tilted fracture, which is consistent with the fracture characteristics of the actual rock specimen. Figure 10a–f shows the horizontal displacement distribution characteristics of specimens with large and small sand particles, respectively. By comparison, it can be seen that with the change of the pore position, the single area of the region with a large displacement value under the condition of large particle size was larger, but the number of distributions was smaller, showing the non-uniformity of the displacement field. When the particle size was smaller, the area of the larger displacement area was smaller, but the number of distributions was larger, showing the uniformity of the displacement field distribution. The heterogeneity of the displacement field directly affected the stability of the specimen. The more non-uniform the distribution of the displacement field, the more likely the specimen is to be damaged. Therefore, it can be concluded that under the condition of large particle size, the deformation of the specimen was non-uniform, and there was a greater chance of breaking.

In order to quantitatively describe the specimen deformation, the maximum horizontal positive, negative displacements, and the maximum difference of the horizontal displacement of the two groups of specimens were collected and summarized. As shown in Table 4, the average value of the maximum positive displacement of the specimen with large particle size was about 15.05 × 10^−3^ mm, while that of the specimen with small particle size was significantly smaller at 10.59 × 10^−3^ mm. For the specimen with large particle size, the average value of the maximum negative displacement was −6.82 × 10^−3^ mm, while that of the specimen with small particle size was about −4.14 × 10^−3^ mm. The difference between the extreme values of the horizontal displacement of the two represents the degree of deformation. The deformation range of the specimen was about 21.87 × 10^−3^ mm under the condition of large particle size, while it was significantly smaller (13.11 × 10^−3^ mm), under the condition of small particle size. It can be seen that the degree and range of deformation of specimens with large particle sizes were larger than those of specimens with small particle size, so they were more likely to be broken.

#### 5.3.2. Analysis of the Force of the Specimen

**Figure 11:**
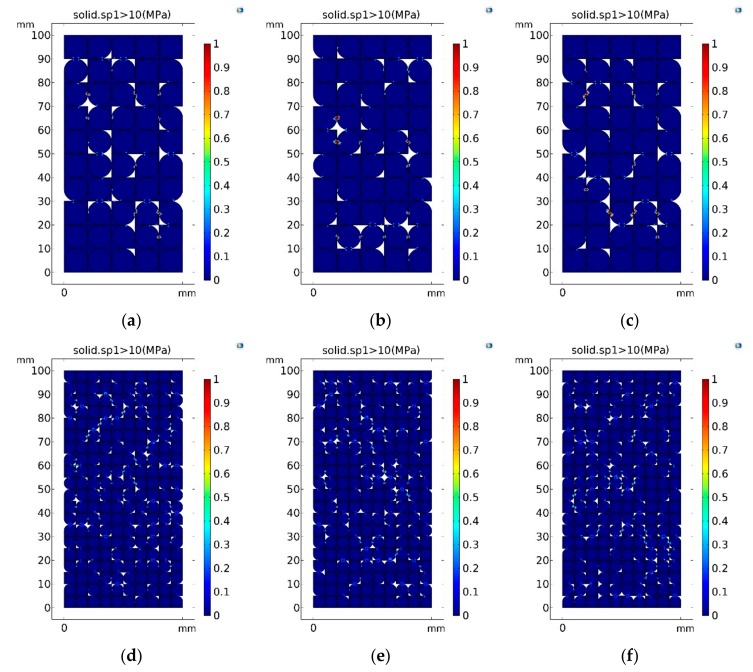
Distribution characteristics of ultimate tensile strength area. (**a**–**c**) The maximum principal stress exceeded 10 MPa of large particle samples in three random simulation experiments; (**d**–**f**) the maximum principal stress exceeded 10 MPa of small particle samples in three random simulation experiments.

The major form of failure of the specimen after compression deformation is tensile failure. Therefore, the distribution of tensile stresses can be used to determine the probability of failure of the specimen under different particle size conditions. According to previous studies, the tensile strength of gypsum hydration reaction cement is 6.5–11.5 MPa [28]. This simulation used 10 MPa as the ultimate tensile strength of gypsum cement. Figure 8 shows the distribution of the area where the maximum principal stress in the selected specimen was tensile and exceeded 10 MPa. The area with a color close to the value 1 was considered to be the area where tensile failure may occur. It can be seen from Figure 11 that the internal tensile force of the specimen was mainly concentrated in the position with pores. From Figure 11a–c, it can be seen that the single area of the tensile stress concentration area formed under the condition of large particle size was larger and the number of distributions was smaller. The situation under the condition of large particle size was the opposite, as shown in Figure 11d–f. This shows that when the particle size was large, it was more likely to cause uneven distribution of the tensile stress concentration area in the specimen, which had a greater chance of causing the overall failure of the specimen.

In order to quantitatively compare the specimen failure probability under different conditions, the total areas of the ultimate tensile strength in specimens with different particle sizes were summarized and calculated, and results are shown in Table 5. It can be seen that in the three sets of experiments, the sum of the areas that tend to break under the condition of large particle size was always greater than that of smaller particle size. It shows that the deformation and stress distribution of the specimen were more uneven when the particle size was larger, which promotes the violation of the specimen mechanical balance.

## 6. Conclusions

In this paper, the effect of sand particle size on the mechanical strength of gypsum-cemented similar materials was analyzed through physical and numerical experiments. The following conclusions were drawn:

1. The particle size of sand in gypsum cement has a strong effect on the compressive strength of the material. The range of compressive strength deviation from the target value for different ranges of sand particle size is between −16.51% and 49.79%, and the sand particle size has a negative correlation with the specimen uniaxial compressive strength.

2. When the sand particle size is small, the needle-shaped dihydrate gypsum crystals and short, thick, rod-shaped ones are formed after the hydration reaction. They are highly intertwined, and there are many crystalline contact points per unit volume, resulting in higher material strength. When the particle size is large, dihydrate gypsum crystals mostly exist in layered or plate-like forms, with poor interweaving degree, and few crystal contact points per unit volume, resulting in lower strength of the specimen.

3. When the sand particle size is large, the size of single pores on the surface and inside the specimen is larger. The length and width of the interface crevices are both larger than those with smaller sand particle sizes. As a result, the probability of the presence of large defects in the specimen is higher, and thus the specimen is more likely to be damaged. In contrast, specimens with smaller sand particle sizes have a uniform pore distribution and a lower probability of large defects, which yields a larger compressive strength.

4. The permeability coefficient of similar materials increases with sand particle size. Because the porosity of all specimens is the same, it can be proved that the coalescence of pores in the specimen is better for large particle sizes. As a result, when the specimen is compressed, the possibility of fracture extension and mutual penetration is greater, and thus the overall compressive strength of the specimen is certainly reduced.

5. The numerical simulation results strongly indicate that the horizontal displacement distribution of specimen under the condition of large particle size is more non-uniform than that of the small particle size, and the difference between the positive and negative horizontal displacements is larger. At the same time, the distribution of tensile stresses is more concentrated when the particle size is larger, and the total area with stresses reaching the ultimate tensile strength is larger. The non-uniform distribution of these deformations and stresses makes the mechanical environment in the specimen more prone to instability and failure.

This study is focused on the effect of sand particle size on the mechanical properties of similar gypsum cementation materials from the aspects of gypsum hydration reaction mechanism and physical characteristics of sand particle cementation. Meanwhile, the effect of sand particle size on the mechanical properties of similar materials cemented by gypsum is a complex issue, and there are many other influencing factors, including the typical particle size of the cementite [29], the environmental conditions [30,31], and the sand particle gradation [18,32,33,34,35]. Furthermore, the specific values of the numerical experiments cannot correspond to the laboratory test results one by one, and only reveal the mechanical behavior of similar gypsum materials in the process of compression. These factors will be taken into account in the follow-up studies, and the technique of digital image correlation (DIC) can be used for obtaining the compress deformation of the specimen, and verifying the numerical experiment results better.

## Figures and Tables

**Table 1:** Specimen production ratio scheme and raw material particle size.

**Lithology**	**Density g/cm^3^**	**Compressive Strength /MPa**	**Hemihydrate Plaster/Mesh**	**Heavy Calcium Powder/Mesh**	**Washed Sand/Mesh**
Mudstone	1.7	34.6	300–400	300–400	80–100
**Model Density g/cm^3^**	**Model Compressive Strength /kPa**	**Water**	**Hemihydrate Plaster Ratio/%**	**Heavy Calcium Powder Ratio/%**	**Washed Sand Ratio/%**
1.13	100.2	1/9	21	9	70

**Table 2:** Influence of sand particle size on uniaxial compressive strength (kPa).

-	0.1 mm	0.3 mm	0.5 mm	0.7 mm	0.9 mm	1.0 mm	1.2 mm	1.5 mm	2.0 mm	2.5 mm	3.0 mm
3 d	66.89	55.87	44.17	40.17	35.01	34.06	33.07	31.16	26.30	26.22	22.04
7 d	104.59	102.66	100.15	100.04	99.58	99.39	97.30	88.94	85.70	74.87	61.57
10 d	150.01	133.29	124.93	121.89	119.91	117.52	116.19	114.97	102.62	93.00	83.62

**Table 3:** Variation of permeability coefficient of specimens with different sand particle sizes.

Sand Particle Sizes/mm	h_1/_cm	h_2/_cm	H_/_cm	h_i_/cm	t/s	Q/mL	s/cm^2^	j/1	k/×10^−4^ cm/s	Permeability Coefficient Average/ ×10^−4^cm/s
2.5	136.6	100	80	4	3.9	12.0	30	495.750	2.050	1.770
	137.3	100	80	4	4.4	11.4	30	496.625	1.750	
	141.5	100	80	4	5.7	13.0	30	501.875	1.520	
2.0	139.3	100	80	4	5.3	13.0	30	499.125	1.640	1.670
	139.4	100	80	4	5.8	14.5	30	499.250	1.680	
	139.7	100	80	4	5.9	15.0	30	499.625	1.690	
1.5	139.9	100	80	4	6.6	14.0	30	499.875	1.420	1.410
	140.9	100	80	4	6.8	15.0	30	501.125	1.470	
	141.7	100	80	4	7.0	14.0	30	502.125	1.340	
1.0	143.3	100	80	4	7.1	14.0	30	504.125	1.300	1.310
	143.2	100	80	4	7.0	14.0	30	504.000	1.330	
	143.5	100	80	4	7.1	14.0	30	504.375	1.310	
0.5	147.3	100	80	4	10.0	14.0	30	509.125	0.917	0.919
	140.6	100	80	4	8.5	12.0	30	500.750	0.945	
	142.1	100	80	4	11.0	14.5	30	502.625	0.894	
0.1	154.6	110	80	4	23.0	14.5	30	530.750	0.400	0.415
	152.1	110	80	4	21.0	14.0	30	527.625	0.429	
	152.1	110	80	4	21.0	14.0	30	527.625	0.417	

**Table 4:** Variation of horizontal displacement of specimens with different particle sizes (10^−3^ mm).

-	Maximum Horizontal Positive Displacement		Maximum Horizontal Negative Displacement		Maximum Difference of Horizontal Displacements	
-	Large	Small	Large	Small	Large	Small
1	16.34	9.32	−3.73	−6.55	20.07	16.00
2	15.97	10.74	−8.61	−2.58	24.58	13.32
3	12.83	11.70	−8.12	−3.29	20.95	10.00
Average	15.05	10.59	−6.82	−4.14	21.87	13.11

**Table 5:** Total area where tensile stress exceeds the ultimate strength (×10^−5^ mm^2^).

Group #	1	2	3	Average
Specimens with large sand particles	2.14	1.71	2.51	2.12
Specimens with small sand particles	1.68	1.53	1.76	1.67

## References

[1] Zhang D.S., Fan G.W., Wang X.F. (2012). Characteristics and stability of slope movement response to underground mining of shallow coal seams away from gullies. Int. J. Min. Sci. Technol..

[2] Xia Y.C., Zhi J.F., Sun X.Y. (2005). Study on relation between tectonic stress and coalmining subsidence with similar material simulation. J. Coal Sci. Eng..

[3] Tao M., Chen X., Ding Q.Q. (2013). A method for subsidence monitoring of similar material simulation test in coal mining. Adv. Mater. Res..

[4] Yan S.Y., Yang K., Liao B.C. (2013). Experimental study of high mining-induced stress evolution characteristics of downward relieving mining in Paner coal mine. Rock Soil Mech..

[5] Zhang H.W., Han J., Hai L.X., Li M., Qiao H.B. (2013). Study on closed multiple-seam in the ascending mining technology. J. Min. Saf. Eng..

[6] Wang F.T., Zhang C., Zhang X.G., Song Q. (2015). Overlying strata movement rules and safety mining technology for the shallow depth seam proximity beneath a room mining goaf. Int. J. Min. Sci. Technol..

[7] Wen B.A., Yan Y., Dong Y.X. (2012). The application of similar material in the simulation of overlying strata movement in high inclined seam in Dongbaowei mine. Adv. Mater. Res..

[8] Xing P.W., Song X.M., Fu Y.P. (2012). Study on similar simulation of the roof strata movement laws of the large mining height workface in shallow coal seam. Adv. Mater. Res..

[9] Cai F., Liu Z.G. (2012). Research on Similar materials simulation test for protective coal-seams of group B coal-seams of Panyi coal mine of China. Appl. Mech. Mater..

[10] Hu Q., Zhang S., Wen G., Dai L., Wang B. (2015). Coal-like material for coal and gas outburst simulation tests. Int. J. Rock Mech. Min. Sci..

[11] Li J.G., Wu Y., Wang Y.Y., Qin N., Wang W.X. (2016). Experimental study on self-made similar material of soft rock. Key Eng. Mater..

[12] Yang R.S., Zhang Y.F., Yang L.Y., Wu Y.L., Ma J.H. (2013). Study on the mixing proportion test of similar material gypsum. China Min. Mag..

[13] Zhang Q.Y., Li S.C., Guo X.H., Li Y., Wang H.P. (2008). Research and development of new typed cementitious geotechnical similar material for iron crystal sand and its application. Rock Soil Mech..

[14] Chen L.W., Bai S.W. (2006). Proportioning test study on similar material of rockburst tendency of brittle rockmass. Rock Soil Mech..

[15] Zhao P.X., Li S.G., Zhuo R.S., Lin H.F. (2018). Experimental research on the properties of “solid–gas” coupling physical simulation similar materials and testing by computer of gas in coal rock. Wirel. Pers. Commun..

[16] Cheng W.M., Sun L., Wang G., Du W., Qu H. (2016). Experimental research on coal seam similar material proportion and its application. Int. J. Min. Sci. Technol..

[17] Luo F., Yang B.S., Hao B.B., Sun L.H., Fu M.M. (2013). Mechanical properties of similar material under uniaxial compression and the strength error sources. J. Min. Saf. Eng..

[18] Sari D., Pasamehmetoglu A.G. (2005). The effects of gradation and admixture on the pumice lightweight aggregate concrete. Cem. Concr. Res..

[19] Bosiljkov V.B. (2003). SCC mixes with poorly graded aggregate and high volume of limestone filler. Cem. Concr. Res..

[20] Ke X., Hou H., Zhou M., Wang Y., Zhou X. (2015). Effect of particle gradation on properties of fresh and hardened cemented paste backfill. Constr. Build. Mater..

[21] Jin Y., Han L., Meng Q., Ma D., Han G., Gao F., Wang S. (2018). Experimental investigation of the mechanical behaviors of grouted sand with UF-OA grouts. Processes.

[22] Jia J.X., Li T.B., Cao H.Y., Pei M.S. (2016). Influence of the sand particle size on the compressive strength in the analog model study. Soil Eng. Found..

[23] Lin H.F., Zhai Y.L., Li S.G., Zhao P.X., Li L. (2015). Experimental study on influential factors of physical and mechanics parameters of similar material for new type rock. J. Xi’an Univ. Sci. Technol..

[24] Wu J.Y., Feng M.M., Xu J.M., Qiu P., Wang Y., Han G.S. (2018). Particle size distribution of cemented rockfill effects on strata stability in filling mining. Minerals.

[25] Wu J.Y., Feng M.M., Mao X.B., Xu J.M., Zhang W.L., Ni X.Y., Han G.S. (2018). Particle size distribution of aggregate effects on mechanical and structural properties of cemented rockfill: Experiments and modeling. Constr. Build. Mater..

[26] Börgesson L., Johannesson L.E., Gunnarsson D. (2003). Influence of soil structure heterogeneities on the behaviour of backfill materials based on mixtures of bentonite and crushed rock. Appl. Clay Sci..

[27] Li H.Z., Guo G.L., Zha J.F. (2017). Study on time-varying Characteristics of similar material model strength and the regulation measures. Environ. Earth Sci..

[28] Rao Y.C., Li G.H. (2014). Laboratory determination the tensile strength of modified desulfurization gypsum. Sichuan Build. Mater..

[29] Fall M., Benzaazoua M., Saa E.G. (2008). Mix proportioning of underground cemented tailings backfill. Tunn. Undergr. Space Technol..

[30] Fall M., Célestin J.C., Pokharel M., Touré M. (2010). A contribution to understanding the effects of curing temperature on the mechanical properties of mine cemented tailings backfill. Eng. Geol..

[31] Yilmaz E., Belem T., Benzaazoua M. (2014). Effects of curing and stress conditions on hydromechanical, geotechnical and geochemical properties of cemented paste backfill. Eng. Geol..

[32] Garcia C., Trendafilova I., Zucchelli A. (2018). The Effect of Polycaprolactone Nanofibers on the Dynamic and Impact Behavior of Glass Fibre Reinforced Polymer Composites. J. Compos. Sci..

[33] Garcia C., Trendafilova I., Zucchelli A., Contreras J. (2018). The effect of nylon nanofibers on the dynamic behaviour and the delamination resistance of GFRP composites. MATEC Web Conf..

[34] Wu J.Y., Feng M.M., Ni X.Y., Mao X.B., Chen Z.Q., Han G.S. (2019). Aggregate gradation effects on dilatancy behavior and acoustic characteristic of cemented rockfill. Ultrasonics.

[35] Gautam B.P., Panesar D.K., Sheikh S.A., Vecchio F.J. (2017). Effect of coarse aggregate grading on the ASR expansion and damage of concrete. Cem. Concr. Res..

